# Can a 1-Item Scale for Psychotherapy Outcomes Be Psychometrically Robust?

**DOI:** 10.32872/cpe.15207

**Published:** 2025-02-28

**Authors:** Scott T. Meier

**Affiliations:** 1 Counseling, School and Educational Psychology, University at Buffalo, Buffalo, NY, USA

Gonçalves et al. (2024) recently described the selection of a 1-item outcome scale for the European Psychotherapy Consortium. The field has been trending toward brief scales because of research indicating greater patient compliance with fewer items (Miller et al., 2003; Miller et al., 2005). From a psychometric perspective, however, the 1-item emotional and psychological outcomes (EPO-1) measure is likely to produce low reliability and validity estimates. This results from measurement principles indicating that (a) reliability estimates increase with the number of items, and (b) validity estimates depend upon reliability. Measurement error decreases with an increasing number of item responses because random error sources tend to balance or cancel (Meier, 2013). If a patient misunderstands a question, for example, this error becomes a major influence on data in a single item self-report. Because multiple factors typically influence responses to any psychological item, scores on 1-item scales are less likely to be sensitive to change resulting from psychotherapy than a multi-item scale that aggregates change-relevant variance (Meier, 1997).

Other research suggests that many patients will not interpret the EPO-1 as test developers intended (Schwarz, 1999). Labeling this problem as *intracategory variability*, Dohrenwend (2006) observed that test-takers respond to item content on a self-report measure based on a wide range of personal experiences. When asked to report on a recent serious illness, for example, respondents will describe episodes that vary from simple flu to heart attacks. As a result, the basis on which individuals respond to health-related categories on self-report measures can range “from *the catastrophic* to *the trivial*” (Dohrenwend, 2006, p. 479). The EPO-1’s content is “At this moment, how well do you feel you are getting along emotionally and psychologically?” Patients respond on a 5-point scale ranging from 0 ("Very poorly; I can barely manage to deal with things") to 4 ("Very well; I have no important complaints"). For many individuals, these are cognitively complex tasks likely to lead to heterogeneous response processes and ratings.

Given that single item measures are inappropriate with ambiguous constructs (Allen et al., 2022), future research should evaluate reliability and validity estimates for the EPO-1. At a minimum, EPO-1 scores should evidence (a) stability over time in the absence of any intervention, (b) change over time when the patient participates in a psychosocial intervention, and (c) moderate to high correlations with existing measures of outcome. If EPO-1 scores fail to meet these standards, possible next steps include (a) augmenting EPO-1 data with one or more item(s) related to common factors that have been shown to influence outcome and (b) developing a system that minimizes respondent burden. Regarding (a), working alliance would appear to be a strong candidate given that psychotherapy researchers consistently find a modest positive effect of the client/therapist alliance on outcomes (Flückiger et al., 2018). Regarding (b), recent studies suggest that AI could produce outcome information through analysis of text produced by client discourse recorded during therapy sessions as well as clinicians’ unstructured progress notes (Chu et al., 2024).
